# The Effect of a Silver Nanoparticle Polysaccharide System on Streptococcal and Saliva-Derived Biofilms

**DOI:** 10.3390/ijms140713615

**Published:** 2013-06-28

**Authors:** Mara Di Giulio, Soraya Di Bartolomeo, Emanuela Di Campli, Silvia Sancilio, Eleonora Marsich, Andrea Travan, Amelia Cataldi, Luigina Cellini

**Affiliations:** 1 Department of Pharmacy, University “G. d’Annunzio”, Chieti-Pescara, via dei Vestini 31, 66100 Chieti, Italy; E-Mails: m.digiulio@unich.it (M.D.); s.dibartolomeo@unich.it (S.D.); edicampli@unich.it (E.D.); s.sancilio@unich.it (S.S.); cataldi@unich.it (A.C.); 2 Medicine, Surgery and Health Sciences Department, University of Trieste, Piazza dell’Ospitale 1, 34129 Trieste, Italy; E-Mail: emarsich@units.it; 3 Department of Life Sciences, University of Trieste, via Giorgieri 5, 34127 Trieste, Italy; E-Mail: atravan@units.it

**Keywords:** silver-nanoparticles, polysaccharide, antimicrobial activity, oral *Streptococci*, planktonic and sessile growth, saliva

## Abstract

In this work, we studied the antimicrobial properties of a nanocomposite system based on a lactose-substituted chitosan and silver nanoparticles: Chitlac-nAg. Twofold serial dilutions of the colloidal Chitlac-nAg solution were both tested on *Streptococcus mitis*, *Streptococcus mutans*, and *Streptococcus oralis* planktonic phase and biofilm growth mode as well as on saliva samples. The minimum inhibitory and bactericidal concentrations of Chitlac-nAg were evaluated together with its effect on sessile cell viability, as well as both on biofilm formation and on preformed biofilm. In respect to the planktonic bacteria, Chitlac-nAg showed an inhibitory/bactericidal effect against all streptococcal strains at 0.1% (v/v), except for *S. mitis* ATCC 6249 that was inhibited at one step less. On preformed biofilm, Chitlac-nAg at a value of 0.2%, was able to inhibit the bacterial growth on the supernatant phase as well as on the mature biofilm. For *S. mitis* ATCC 6249, the biofilm inhibitory concentration of Chitlac-nAg was 0.1%. At sub-inhibitory concentrations, the Streptococcal strains adhesion capability on a polystyrene surface showed a general reduction following a concentration-dependent-way; a similar effect was obtained for the metabolic biofilm activity. From these results, Chitlac-nAg seems to be a promising antibacterial and antibiofilm agent able to hinder plaque formation.

## 1. Introduction

Biofilm is characterized by microbial communities that are organized as a network of cell-to-cell interactions [1]. During biofilm formation, microbes aggregate to each other encasing themselves in self-produced extracellular polymer substances generating inside the bacterial sessile population, new conditions of increased antimicrobial tolerance [2,3].

Dental plaque is a complex multispecies biofilm that accumulates on tooth surfaces in the oral cavity; its development and maturation has important implications in the etiology and progression of dental caries and periodontitis that are the most common oral diseases [4]. *Streptococci* are the predominant colonizers of early enamel biofilms and Mitis group *Streptococci*, such as *S. mitis* and *S. oralis*, are the initial colonizers of tooth surface due to their ability to recognize and adhere to the receptors on the acquired pellicle coating the oral surfaces [1]. Their ability to co-aggregate and induce specific cell-cell interaction between genetically distinct microorganisms is crucial to form mature oral biofilm [1]. *S. mutans*, an aciduric bacterium, is one of the most cariogenic bacteria and it is considered the etiological agent of dental caries, responsible of the enamel and dentin demineralization by means of its organic acids production ability [5].

The need for safe, efficacious and economical schemes of alternative prevention as well as new treatments and products for oral diseases is due to the increase of these pathogens in developing countries, and to the wide microbial resistance to commonly used antimicrobial agents in developed areas [6].

During the last few decades, there have been many studies about the antimicrobial activity of polymeric materials [7]. A great contribution to the improvement of the innovative therapies for biofilm-related diseases is given by nanoscale systems because of their tunable size, increased suspendibility, and surface tailorability, which enhance interactions with biological systems at the molecular level [8,9]. Nanotechnology is an emerging field with numerous applications ranging from bioengineering to nano-devices, cosmetics, medicine, and drugs [10,11].

In the microbiological field, nanoparticles possess attractive properties, in particular metallic nanoparticles are the most promising because they show good antibacterial properties due to their large surface area to volume ratio [12]. Several studies have shown that silver ions are able to make structural changes in the cell membrane [13]. Silver has a high affinity for negatively charged side groups on biological molecules such as sulphydryl, carboxyl, phosphate, and other charged groups distributed throughout microbial cells. Silver ions inhibit a number of enzymatic activities by reacting with electron donor groups, especially sulfhydryl groups [14,15]. Silver ions induce the inactivation of critical physiological functions such as cell wall synthesis, membrane transport, nucleic acid (RNA and DNA) synthesis and translation, protein folding and function, and electron transport [13,14,16]. For these reasons “nanosilver” has gained popularity in consumer products. For example, toothbrushes and cosmetics are equipped with silver to obtain a natural antibacterial effect [17]. Silver-based nanotechnologies seem to be effective against biofilms. Recent studies showed that silver is effective against pathogens capable to form biofilm produced by *Pseudomonas aeruginosa*, *S. oralis*, *Staphylococcus epidermidis*, and some works document the effect of silver nanoparticles on oral microorganism and on dental microbial biofilm [18,19].

A crucial issue concerning silver nanoparticles is their tendency to form aggregates, losing their peculiar properties associated with the nanoscale, polyelectrolytes in small concentration, such as polyphosphate, polyacrylate, poly(vinyl-sulfate), poly(ethylene-imine), poly(allyl-amine), and chitosan, have been used to stabilize the nanoparticles to prevent the aggregates formation [20,21].

Chitosan, a polysaccharide biopolymer from natural sources, has been proposed for use in a number of applications such as wound healing, food packaging for its special biocompatible, antimicrobial and antibiofilm properties [22] as well as its versatility to be formulated into nanoparticles, fiber or film and membranes. It has also been used to prepare and stabilize metal nanoparticles [23]. Unfortunately, chitosan presents a solubility limitation, it can be solubilized only at low pH and it is insoluble at neutral pH conditions. This aspect was improved by synthesizing a lactose-substituted chitosan, 1-deoxylactit-1-yl chitosan, short-named “Chitlac”. Chitlac is a highly branched biocompatible and bioactive polymer, owing to the terminal galactose unit on the side chain. A new solution composed by silver nanoparticles in the bioactive Chitlac was recently investigated for its bactericidal and non-cytotoxic effects and named Chitlac-nAg [20,24,25].

In the present work, we investigated the antimicrobial properties of Chitlac-nAg, on planktonic and sessile phases of oral *Streptococci*. In particular, both free-living cells and in formation and mature biofilms of *S. mitis*, *S. mutans*, and *S. oralis* were analyzed. Moreover, the effect of Chitlac-nAg was also studied on saliva samples, both on planktonic and sessile growth mode.

## 2. Results and Discussion

### 2.1. Results

The effects of different Chitlac-nAg concentrations on the planktonic and sessile growth phases of *Streptococcus* strains were shown in Table 1. In respect to the planktonic bacteria, the Chitlac-nAg showed an interesting bactericidal effect against all streptococcal strains at 0.1% (v/v) and this value represented also the minimum inhibitory concentration, except for *S. mitis* ATCC 6249 that was inhibited at one step less.

A value of 0.2% (v/v) of Chitlac-nAg was able to inhibit the bacterial growth both on the fluid supernatant of the streptococcal biofilms (BIC) and on the mature biofilm (BEC). Against *S. mitis* ATCC 6249, Chitlac-nAg expressed its antibiofilm effect with a BIC value of 0.1% (v/v).

**Figure 1:**
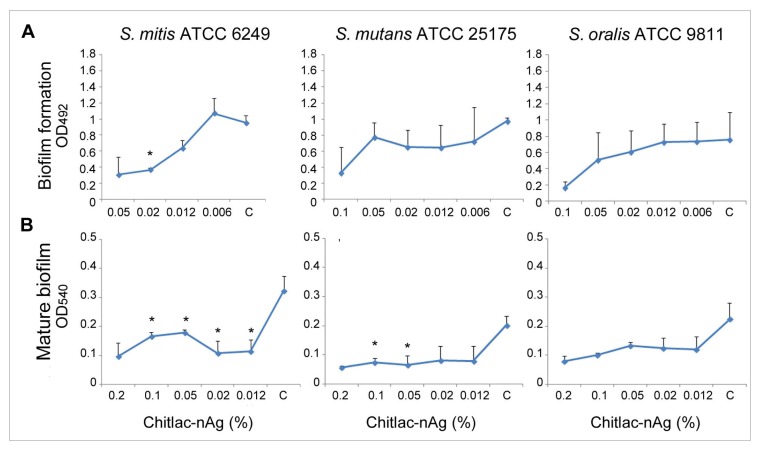
Effect of Chitlac-nAg on *Streptococcus* strains. (**A**) Biofilm formation, (**B**) Mature biofilm. (C = control). Values represent the mean (±SD) of three independent experiments performed in duplicate. Asterisks indicate the values with a significant reduction in comparison to the respective control.

The analysis of the Chitlac-nAg effect at sub-inhibitory concentrations on the biofilm formation showed a general reduction of the adhesion of the Streptococcal strains on a polystyrene surface in an Chitlac-nAg concentration-dependent-way which was significant (*p* < 0.05) in *S. mitis* ATCC 6249 (Figure 1A).

A similar effect was obtained on the mature biofilm through the reduction of the metabolic activity of cells embedded into the matrix (Figure 1B). In particular, each sub-inhibitory Chitlac-nAg concentration was effective in the reduction of metabolic activity of *S. mitis* ATCC 6249 mature biofilm (*p* < 0.05) with respect to the control. Significant metabolic decreases of *S. mutans* ATCC 25175 mature biofilm were recorded at 0.1% and 0.05% (v/v) of Chitlac-nAg.

**Figure 2:**
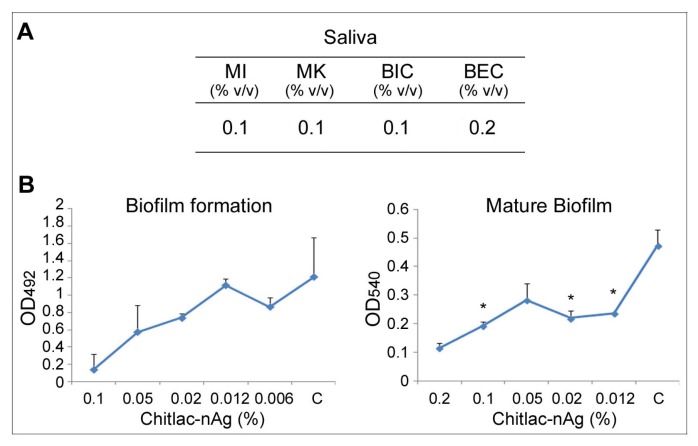
Effect of Chitlac-nAg on saliva. (**A**) Antimicrobial evaluation against planktonic phase (MI, MK) and sessile phase (BIC, BEC); (**B**) Effect of sub-inhibitory concentrations of Chitlac-nAg on biofilm formation and on mature biofilm. The values (±SD) are the mean of three independent experiments performed in duplicate. (MI = microbial inhibition, MK = microbial killing, C = control). Asterisks indicate the values with a significant reduction in comparison to the respective control.

To mimic the oral microbial complexity, the Chitlac-nAg effect was also evaluated both on the saliva planktonic phase, and on the saliva-derived biofilm (Figure 2).

Chitlac-nAg showed a general interesting antimicrobial effect against saliva mixed microorganisms. At the same concentration of 0.1% (v/v), Chitlac-nAg was able both to inhibit/kill salivary microorganisms in the planktonic phase, and to inhibit the saliva-derived biofilm (Figure 2A).

At sub-inhibitory concentrations, the biofilm formation capability of the salivary microrganisms on a polystyrene surface was progressively reduced, this effect was more relevant in the mature biofilm. Significant differences were obtained on the evaluation of microbial biofilm metabolic activity (MTT assay) at 0.012%, 0.02% and 0.1% Chitlac-nAg concentrations (Figure 2B), with respect to the control.

### 2.2. Discussion

This study evaluates the effect of a new nanocomposite system based on silver nanoparticles against oral microorganisms in planktonic and sessile growth. This system combines a biocompatible lactose-modified chitosan: “Chitlac” with the antibacterial properties of silver at the nanoscale level.

This system has been previously tested for its non-cytotoxic effect towards different eukaryotic cell lines and for its antimicrobial activity against *S. epidermidis* and *P. aeruginosa* [20,24,25].

This novel approach facilitates the use of silver nanoparticles—biopolymer composites in the preparation of bioactive biomaterial useful in the oral cavity. In this environment, oral streptococci represent the first colonizers, in fact they constitute more than 70% of the oral microflora [26]. In particular, *S. mutans* strains are known as causative of oral caries and sometimes are able to cause infective endocariditis and bacteriemia [27]. Moreover, *S. mitis* strains, usually found as a commensal of human oral environment, can exert a strong immunomodulatory effect and a protective effect on human cells, and can cause a variety of infectious complications including infective endocarditis, bacteriemia and septicaemia [28–30].

Biofilms are well-organized communities of microorganisms that colonize surfaces in a humidified environment and, undoubtedly, the polymicrobial oral biofilm is the most well-known example [1]. The persistence of microorganisms within oral cavity is due to their capability to adhere to the surfaces (tooth and mucosal surfaces) and to organize themselves in a biofilm constituting the normal flora of the human oral cavity. When the oral microecology is locally disturbed, bacteria could cause severe diseases as dental caries, periodontal diseases, and also induce endodontic and orthodontic infections [31]. Biofilm-related infections are difficult to treat because they are tolerant to the conventional chemotherapeutics, so innovative therapies are required to hinder their formation and to promote their eradication [3]. As Kolebrander underlined, oral diseases may be influenced by microbial communities, not by single pathogens highlighting as the community becomes the etiological agent [1].

In this study, Chitlac-nAg at very low concentrations, provided the inhibition of the growth of the planktonic streptococcal population tested. Interestingly, the concentration effective in the inhibition of the bacterial growth was also capable to obtain the cellular killing, and a step higher was efficacious against their sessile counterpart.

A similar behavior was also recorded by studying the antimicrobial effect of this new compound against saliva. We include the whole saliva in this study because in the mouth a biofilm single species does not exist, so to better reproduce an oral microcosm, we detected whole saliva as inoculum.

The effective Chitlac-nAg results on oral microorganisms may be attributed to the higher surface area and charge density that makes the Ag-nanoparticles able to obtain a major interaction with the negatively charge of cell surface; as proposed by Morones *et al.* (2005), silver ions are able to interact with sulfate-groups of some cellular enzymes and with phosphorus-groups of molecules as nucleic acids. These effects could induce a modification in the cellular permeability and lead the cells to death [14]. The Chitlac-nAg antibacterial effect can be ascribed only to the properties of silver nanoparticles since the functionalization of chitosan with lactose leads to the loss of its antibacterial activity, but allows the stabilization of the colloidal solution [25].

Together with the silver antibacterial property, the small size of nanoparticles enables them to penetrate through the biofilm matrix and to reach bacterial cells.

Moreover, at sub-MIC values, Chitlac-nAg affects the biofilm forming capability of all strains studied suggesting a progressive cell death or a progressive modification on the cell surface that makes bacteria unable to adhere on polystyrene surfaces.

From our results we can assert that Chitlac-nAg seems to be a promising silver nanoparticles colloidal system for antibacterial and antibiofilm treatment in the oral cavity. Chitlac-nAg possesses a potential antibiofilm capability to reduce biofilm and contrast its formation. This property could be useful to prevent the biofilm formation on restorative materials, and to avoid secondary caries, or to complex Chitlac-nAg to toothpaste or composite to contrast oral hygiene-related diseases.

## 3. Experimental Section

### 3.1. Bacterial Strains and Saliva Collection

The reference strains: *Streptococcus mitis* ATCC6249*, S. mutans* ATCC 25175, and *S. oralis* ATCC 9811 were used in this study. The strains were stored at −80 °C in Microbanks (Pro-Lab Diagnostics) and a single bead was removed from the cryovials and inoculated directly into Tryptic Soy Broth (TSB; Oxoid Milan, Italy) at 37 °C for 18–24 h under an anaerobic atmosphere, for the overnight cultures.

Whole saliva was collected from a healthy adult donor, following a previous study [32]. The donor had natural dentition without active caries or periodontal pathology, and without the use of antibiotics within the last 3 months. The donor abstained from food/drink intake for 2 h prior to donating saliva, unstimulated saliva was collected by spitting method in polypropylene tube and was kept on ice. The whole fresh saliva was centrifuged at 2500*g* for 10 min to remove debris and the supernatant was used as inoculum for each experiment to reproduce the typical microbial composition occurring in the oral cavity.

### 3.2. Determination of the Minimum Inhibitory Concentration (MIC) and Minimum Bactericidal Concentration (MBC)

Chitlac-silver nanoparticles (Chitlac-nAg) were prepared as previously shown [20]. Briefly, AgNO_3_ and C_6_H_8_O_6_ were added to a Chitlac solution (2 g/L) to give final concentrations of 1 mM (silver nitrate) and 0.5 mM (ascorbic acid). The MIC and MBC were measured according to EUCAST guidelines with some modifications [33]. The MIC is defined as the lowest concentration of an antimicrobial agent that completely inhibits visible bacterial growth, and the MBC is defined as the lowest concentration of an antimicrobial agent that kills 99.9% of the initial inoculum. Each bacterial overnight culture was diluted in 30% TSB to give 0.5 McFarland, and then diluted 1:100 in the same medium to obtain 5 × 10^5^ CFU per well.

The colloidal Chitlac-nAg solution was diluted two-fold from its stock solution in 30% TSB and 100 μL of each two-fold dilution plus 100 μL of the standardized bacterial culture or saliva (to determine the Microbial Inhibition, MI, value) were added into each well of a 96-well tissue culture plate (tissue-culture-treated plates; Nunc, EuroClone SpA, Life-Sciences-Division, Milano, Italy). To control, an equivalent volume of 30% TSB was added to the bacterial cultures instead of Chitlac-nAg dilutions. The plates were incubated at 37 °C for 48 h with 5% CO_2_. After the MIC reading for the MBC measurement, 100 μL aliquots from wells without visible growth were spread on Tryptic Soy Agar plates (TSA, Oxoid Milan, Italy) and incubated at 37 °C for 24 h with 5% CO_2_.

For the saliva sample, to determine the microbial killing (MK) value, 100 μL aliquots from wells without visible growth were spread on TSA plates and incubated at 37 °C for 24 h with 5% CO_2_.

All experiments were performed in duplicate for three independent experiments.

### 3.3. Chitlac-nAg Effect on Biofilm Formation

The effect of different concentrations of Chitlac-nAg (ranging from MIC to 0.125 MIC) on Streptococcal and whole saliva biofilm-forming ability was tested on polystyrene flat-bottomed microtitre plates, as previously described [34]. Briefly, streptococcal cultures were grown overnight in TSB, diluted in TSB 30% plus 1% sucrose to 0.5 McFarland and 100 μL was dispensed into each well of 96-well polystyrene flat-bottomed microtitre plates in the presence of 100 μL subinhibitory concentrations (subMIC) of Chitlac-nAg (diluted in 30% TSB plus 1% sucrose) or 100 μL TSB 30% plus 1% sucrose (control). After incubation for 48 h at 37 °C at 5% CO_2_, each well was washed twice with sterile PBS (pH 7.4), dried, stained for 1 min with 0.1% safranin, and washed with water. The stained biofilms were resuspended in 200 μL ethanol, and OD_492_ was measured by spectrophotometry using an ELISA reader.

Each assay was performed in duplicate for three independent experiments.

### 3.4. Chitlac-nAg Effect on Mature Biofilm

Streptococcal strains, as well as the whole saliva, were grown as biofilms using polystyrene flat-bottomed microtitre plates in TSB plus 1% sucrose for bacteria and at 1% sucrose for saliva. After 24 h of incubation at 37 °C at 5% CO_2_, the planktonic-phase cells were gently removed and the wells were washed with PBS and filled with 200 μL twofold dilutions of the Chitlac-nAg in 30% TSB plus 1% sucrose, or 30% TSB plus 1% sucrose (control). The plates were incubated for 24 h at 37 °C at 5% CO_2_. The OD_600_ was measured at time 0 and after incubation for 24 h by spectrophotometry using an ELISA reader. The biofilm inhibitory concentration (BIC) was determined as the lowest concentration where no growth occurred in the supernatant fluid, confirmed by no increase in optical density compared with the initial reading. For the determination of the biofilm eradication concentration (BEC), samples of biofilms from the bottom of these wells were scraped by a metal loop, and spread on TSA plates and incubated for 48 h at 37 °C at 5% CO_2_. The BEC value was determined as the lowest concentration at which no bacterial growth occurred on the TSA plates.

Each assay was performed in duplicate for three independent experiments.

### 3.5. MTT Assay of Metabolic Biofilm Activity

The MTT (3-[4,5-dimethylthiazol-2-yl]-2,5-diphenyltetrazolium bromide) assay is a colorimetric assay in which the yellow tetrazole, MTT, is reduced to purple formazan by living, respiratory active cells [35]. The Streptococci and whole saliva were grown as biofilms as described above, after the planktonic-phase cells removing, the wells were washed with PBS and filled with 200 μL twofold dilutions of the Chitlac-nAg in 30% TSB plus 1% sucrose, or 30% TSB plus 1% sucrose (control) for 24 h at 37 °C at 5% CO_2_. After Chitlac-nAg treatment, 200 μL of MTT dye (0.5 mg/mL MTT in PBS) was dispensed into each well of microtiter plates and incubated at 37 °C in 5% CO_2_ for 1 h. During this time, metabolically active bacteria reduced the MTT to purple formazan. After 1 h, 200 μL of dimethyl sulfoxide (DMSO) was added to solubilize the formazan crystals, and the plate was incubated for 20 min with gentle mixing at room temperature in the dark. After mixing by pipetting, 200 μL of the DMSO solution from each well was transferred to a 96-well plate, and the OD_540_ was measured by ELISA reader. A higher absorbance is related to a higher formazan concentration, which indicates a higher metabolic activity in the biofilm.

Each assay was performed in duplicate for three independent experiments.

### 3.6. Statistical Analysis

The significance of differences between controls and experimental group was evaluated using Student’s *t*-test. *p* values < 0.05 indicate that the recorded differences were considered statistically significant.

## 4. Conclusions

In conclusion, Chitlac-nAg seems to be a promising antibacterial and antibiofilm agent able to hinder the plaque formation. Additional studies need to be carried out to better recommend the use of Chitlac-nAg *in vivo* to prevent secondary caries and the bacteria/pathogens adhesion on medical devices.

## Figures and Tables

**Table 1:** Antibacterial and anti-biofilm effects of Chitlac-nAg on *Streptococcus* strains.

Streptococcal strains	MIC (% v/v)	MBC (% v/v)	BIC (% v/v)	BEC (% v/v)
*S. mitis* ATCC 6249	0.05	0.1	0.1	0.2
*S. mutans* ATCC 25175	0.1	0.1	0.2	0.2
*S. oralis* ATCC 9811	0.1	0.1	0.2	0.2

MIC: minimum inhibitory concentration; MBC: minimum bactericidal concentration; BIC: biofilm inhibitory concentration; BEC: biofilm eradication concentration.

## References

[1] Kolenbrander P.E. (2011). Multispecies communities: Interspecies interactions influence growth on saliva as sole nutritional source. Int. J. Oral Sci.

[2] Lewis K. (2012). Persister cells: Molecular mechanisms related to antibiotic tolerance. Handb. Exp. Pharmacol.

[3] Römling U., Balsalobre C. (2012). Biofilm infections, their resilience to therapy and innovative treatment strategies. J. Intern. Med.

[4] Filoche S., Wong L., Sissons C.H. (2010). Oral biofilms: Emerging concepts in microbial ecology. J. Dent. Res.

[5] Napimoga M.H., Höfling J.F., Klein M.I., Kamiya R.U., Gonçalves R.B. (2005). Tansmission, diversity and virulence factors of *Streptococcus mutans* genotypes. J. Oral Sci.

[6] Petersen P.E., Bourgeois D., Ogawa H., Estupinan-Day S., Ndiaye C. (2005). The global burden of oral diseases and risks to oral health. Bull. World Health Organ.

[7] Muñoz-Bonilla A., Fernández-García M. (2012). Polymeric materials with antimicrobial activity. Prog. Polym. Sci.

[8] Iannitelli A., Grande R., Di Stefano A., Di Giulio M., Sozio P., Bessa L.J., Laserra S., Paolini C., Protasi F., Cellini L. (2011). Potential antibacterial activity of carvacrol-loaded poly(*DL*-lactide-co-glycolide) (PLGA) nanoparticles against microbial biofilm. Int. J. Mol. Sci.

[9] Mahapatro A., Singh D.K. (2011). Biodegradable nanoparticles are excellent vehicle for site directed *in vivo* delivery of drugs and vaccines. J. Nanobiotechnol.

[10] Loh X.J., Lee T.C. (2012). Gene delivery by functional inorganic nanocarriers. Recent Pat. DNA Gene Seq.

[11] Gupta A.S. (2011). Nanomedicine approaches in vascular disease: A review. Nanomedicine.

[12] Azam A., Ahmed A.S., Oves M., Khan M.S., Habib S.S., Memic A. (2012). Antimicrobial activity of metal oxide nanoparticles against Gram-positive and Gram-negative bacteria: A comparative study. Int. J. Nanomed.

[13] Jung W.K., Koo H.C., Kim K.W., Shin S., Kim S.H., Park Y.H. (2008). Antibacterial activity and mechanism of action of the silver ion in *Staphylococcus aureus* and *Escherichia coli*. Appl. Environ. Microbiol.

[14] Morones J.R., Elechiguerra J.L., Camacho A., Holt K., Kouri J.B., Ramirez J.T. (2005). The bactericidal effect of silver nanoparticles. Nanotechnology.

[15] Rai M., Yadav A., Gade A. (2001). Silver nanoparticles as a new generation of antimicrobials. Biotechnol. Adv..

[16] Al-Ahmad A., Wiedmann-Al-Ahmad M., Deimling D., Jaser C., Pelz K., Wittmer A., Ratka-Krüger P. (2010). An antimicrobial effect from silver-coated toothbrush heads. Am. J. Dent.

[17] Masurkar S.A., Chaudhari P.R., Shidore V.B., Kamble S.P. (2012). Effect of biologically synthesised silver nanoparticles on *Staphylococcus aureus* biofilm quenching and prevention of biofilm formation. IET Nanobiotechnol.

[18] Cabal B., Cafini F., Esteban-Tejeda L., Alou L., Bartolomé J.F., Sevillano D., López-Piriz R., Torrecillas R., Moya J.S. (2012). Inhibitory effect on *in vitro Streptococcus oralis* biofilm of a soda-lime glass containing silver nanoparticles coating on titanium alloy. PLoS One.

[19] Kalishwaralal K., BarathManiKanth S., Pandian S.R., Deepak V., Gurunathan S. (2010). Silver nanoparticles impede the biofilm formation by *Pseudomonas aeruginosa* and *Staphylococcus epidermidis*. Colloids Surf. B.

[20] Travan A., Pelillo C., Donati I., Marsich E., Benincasa M., Scarpa T., Semeraro S., Turco G., Gennaro R., Paoletti S. (2009). Non-cytotoxic silver nanoparticle-polysaccharide nanocomposites with antimicrobial activity. Biomacromolecules.

[21] Grunlan J.C., Choi J.K., Lin A. (2005). Antimicrobial behavior of polyelectrolyte multilayer films containing cetrimide and silver. Biomacromolecules.

[22] Kumar M.N., Muzzarelli R.A., Muzzarelli C., Sashiwa H., Domb A.J. (2004). Chitosan chemistry and pharmaceutical perspectives. Chem. Rev.

[23] Huang H., Yuan Q., Yang X. (2004). Preparation and characterization of metal-chitosan nanocomposites. Colloids Surf. B.

[24] Marsich E., Travan A., Donati I., Turco G., Kulkova J., Moritz N., Aro H.T., Crosera M., Paoletti S. (2013). Biological responses of silver-coated thermosets: An *in vitro* and *in vivo* study. Acta Biomater.

[25] Travan A., Marsich E., Donati I., Benincasa M., Giazzon M., Felisari L., Paoletti S. (2011). Silver-polysaccharide nanocomposite antimicrobial coatings for methacrylic thermosets. Acta Biomater.

[26] Damian M., Palade A.M., Băltoiu M., Petrini A., Păuna M., Roseanu A. (2010). Phenotypic and molecular methods used for identification of oral streptococci and related microorganisms. Roum. Arch. Microbiol. Immunol.

[27] Bowen W.H., Koo H. (2011). Biology of *Streptococcus mutans*-derived glucosyltransferases: Role in extracellular matrix formation of cariogenic biofilms. Caries Res.

[28] Mitchell J. (2011). *Streptococcus mitis*: Walking the line between commensalism and pathogenesis. Mol. Oral Microbiol.

[29] Di Giulio M., D’Ercole S., Zara S., Cataldi A., Cellini L. (2012). *Streptococcus mitis*/human gingival fibroblasts co-culture: The best natural association in answer to the 2-hydroxyethyl methacrylate release. APMIS.

[30] Zara S., Di Giulio M., D’Ercole S., Cellini L., Cataldi A. (2011). Anti-adhesive and pro-apoptotic effects of 2-hydroxyethyl methacrylate on human gingival fibroblasts co-cultured with *Streptococcus mitis* strains. Int. Endod. J.

[31] Busscher H.J., Rinastiti M., Siswomihardjo W., van der Mei H.C. (2010). Biofilm formation on dental restorative and implant materials. J. Dent. Res.

[32] Cheng L., Exterkate R.A., Zhou X., Li J., ten Cate J.M. (2011). Effect of *Galla chinensis* on growth and metabolism of microcosm biofilms. Caries Res.

[33] European Committee for Antimicrobial Susceptibility Testing (EUCAST) of the European Society of Clinical Microbiology and Infectious Dieases (ESCMID) (2000). Terminology relating to methods for the determination of susceptibility of bacteria to antimicrobial agents. Clin. Microbiol. Infect..

[34] D’Ercole S., Di Giulio M., Grande R., Di Campli E., Di Bartolomeo S., Piccolomini R., Cellini L. (2011). Effect of 2-hydroxyethyl methacrylate on *Streptococcus* spp. biofilms. Lett. Appl. Microbiol..

[35] Antonucci J.M., Zeiger D.N., Tang K., Lin-Gibson S., Fowler B.O., Lin N.J. (2012). Synthesis and characterization of dimethacrylates containing quaternary ammonium functionalities for dental applications. Dent. Mater.

